# Benchmarking of bioinformatics tools for the hybrid *de novo* assembly of human and non-human whole-genome sequencing data

**DOI:** 10.1016/j.csbj.2025.07.020

**Published:** 2025-07-13

**Authors:** Adrián Muñoz-Barrera, Luis A. Rubio-Rodríguez, David Jáspez, Almudena Corrales, Itahisa Marcelino-Rodriguez, Lourdes Ortiz, Pablo Mendoza, José M. Lorenzo-Salazar, Rafaela González-Montelongo, Carlos Flores

**Affiliations:** a Genomics Division, Instituto Tecnológico y de Energías Renovables (ITER), Santa Cruz de Tenerife, Spain; b Research Unit, Hospital Universitario Nuestra Señora de Candelaria, Instituto de Investigación Sanitaria de Canarias, Santa Cruz de Tenerife, Spain; c CIBER de Enfermedades Respiratorias, Instituto de Salud Carlos III, Madrid, Spain; d Preventive Medicine and Public Health Area, Universidad de La Laguna, San Cristóbal de La Laguna, Spain; e Institute of Biomedical Technologies, Universidad de La Laguna, San Cristóbal de La Laguna, Spain; f Department of Research and Development in Molecular Diagnostic, Vircell S.L., Granada, Spain; g Plataforma Genómica de Alto Rendimiento para el Estudio de la Biodiversidad, Associated Unit to Consejo Superior de Investigaciones Científicas (CSIC) by Instituto de Productos Naturales y Agrobiología (IPNA), San Cristóbal de La Laguna, Spain; h Facultad de Ciencias de la Salud, Universidad Fernando de Pessoa Canarias, Las Palmas de Gran Canaria, Spain

**Keywords:** Long-read sequencing, Nanopore, WGS, De novo genome assembly

## Abstract

Accurate and complete *de novo* genome assemblies enable variant identification and the discovery of novel genomic features and biological functions. However, *de novo* assemblies of large and complex genomes remain challenging. Long-read sequencing data, alone or combined with short-read data, facilitate genome assembly. However, the literature has limited comprehensive evaluations of software performance, especially for human genome assembly. We benchmarked 11 pipelines, including four long-read only assemblers and three hybrid assemblers, combined with four polishing schemes, using the HG002 human reference material sequenced with Oxford Nanopore Technologies and Illumina. The best-performing pipeline was validated with non-reference human and non-human routine laboratory samples. Software performance was assessed using QUAST, BUSCO, and Merqury metrics, alongside computational cost analyses. We found that Flye outperformed all assemblers, particularly with Ratatosk error-corrected long-reads. Polishing improved the assembly accuracy and continuity, with two rounds of Racon and Pilon yielding the best results. The assembly of data from validation samples showed comparable assembly metrics to those of the reference material. Based on the results, a complete optimal analysis pipeline for the assembly, polishing, and contig curation developed on Nextflow is provided to enable efficient parallelization and built-in dependency management to further advance the generation of high-quality and chromosome-level assemblies.

## Graphical Abstract

**Figure:**
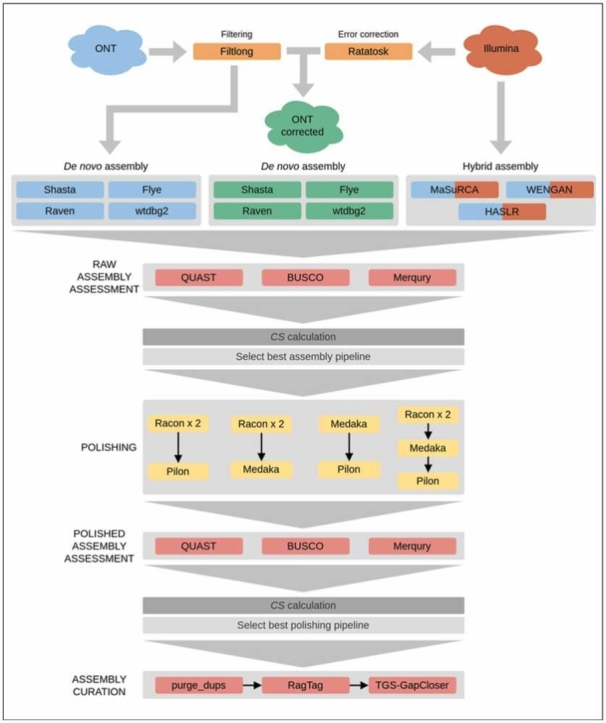

## 1 Introduction

Next-generation sequencing technologies (NGS) have enabled us to rapidly expand our knowledge of the human genome with unprecedented precision [1]. As the Precision Medicine paradigm shift is embraced, the accurate reconstruction of individual human genomes is key, necessitating the deployment of robust bioinformatics tools for *de novo* assembly [2] to provide a comprehensive and unbiased understanding of a patient’s DNA sequence. *De novo* genome assembly is crucial for unveiling the full spectrum of genetic diversity among individuals, shedding light on population-specific variation [3], [4], [5] and rare alleles that may influence disease risk and treatment response [6], [7], among other applications. Third-generation sequencing (TGS), as seen in Oxford Nanopore Technologies (ONT) and Pacific Biosciences (PacBio), relies on long-read capabilities that boost the possibilities of a comprehensive view of the genome [8]. Traditional short-read NGS retains optimal turnaround time and cost-effectiveness. However, the existing algorithms struggle to provide a highly continuous *de novo* genome assembly based on short reads, failing to resolve complex genomic regions, repetitive elements, and most of the structural variants. Despite the lower per base accuracy compared to short-read NGS, TGS is key for solving these problems [9], [10], especially in humans, since a significant portion of the genome consists of repetitive sequences and many of them are involved in disease causes [11].

Unlike others, ONT reads typically span thousands of bases [12] providing a more comprehensive and contiguous view of genomic regions that are otherwise challenging to resolve [13], [14]. To harness the benefits of ONT long-reads while addressing their inherent error profiles, two primary strategies are typically employed. One involves error correction of long reads before the actual genome assembly, which is achieved with high coverage sequencing [15], [16], [17]. For this, information from multiple reads covering the same genomic region is leveraged to identify and rectify random errors, thereby enhancing the reliability of the raw long-read data. Another strategy polishes the draft assembled sequence obtained from long reads [18], so that the draft genome sequence undergoes iterative refinement through alignment with high-quality short-read data or consensus sequences. This step helps to rectify any remaining errors, fine-tuning the accuracy and completeness of the assembled genome. The combination of pre-assembly error correction and post-assembly polishing ensures a more robust and accurate representation of the genome [19], [20], [21].

In parallel, the PacBio HiFi technology has gained attention since it can generate long and highly accurate reads [22]. Additionally, complementary technologies, such as Hi-C sequencing, have been widely adopted for chromosome-level scaffolding, facilitating the generation of near-complete assemblies by capturing long-range spatial interactions within the genome [23], [24]. Although PacBio HiFi offers exceptional accuracy, its higher cost and infrastructure requirements still limit its wider adoption. In this study we focused on ONT due to its broader accessibility, lower upfront cost, and scalability for real-time and portable sequencing. In combination with Illumina in hybrid assembly approaches, the long-range continuity of ONT data can be effectively integrated with the high accuracy of Illumina reads to enhance overall assembly quality [25].

Despite the importance and the existence of different bioinformatics tools for *de novo* assembly of genomes, there is a lack of studies assessing the benefits and limitations of the available tools for ONT read data, especially in the context of humans [26]. Several studies have assessed the performance of genome assembly tools using different types of sequencing data. Initiatives such as the Genome in a Bottle (GIAB) consortium and the precisionFDA Truth Challenges have provided valuable reference materials and evaluation frameworks for benchmarking variant calling and assembly pipelines [11], [27]. Most studies have primarily focused on comparing short-read or long-read assemblers independently. Worth noting are the studies by Shafin *et al.* [28], evaluating nanopore-only assemblies, and Wenger *et al.* [29], examining PacBio HiFi data. However, there is a lack of benchmarking studies in the literature that systematically evaluate hybrid *de novo* assembly pipelines combining long-read and short-read technologies. In particular, the performance of assembly tools combined with polishing strategies in the context of human whole-genome data has not been thoroughly explored.

Here we aimed to benchmark alternative *de novo* genome assembly bioinformatics tools of nanopore data from human whole-genomes. For that, we used a human reference material sequenced with ONT and Illumina, and then validated the performance of the best benchmarked tool in a non-reference routine laboratory sample with lower integrity. To further validate its performance in datasets obtained with newer nanopore versions and to broaden its applicability in non-human samples, the pipeline was also tested in reconstructing bacterial and viral genomes. Finally, we also contribute with a complete best-performing analysis pipeline for assembly, polishing, and contig curation developed on Nextflow enabling parallelization and built-in dependency management.

## 2 Materials and methods

In brief, the study workflow involved the use of data from a human sample sequenced with ONT and Illumina to benchmark 11 *de novo* and hybrid assembly pipelines, combined with different polishing schemes to improve the accuracy and continuity of the assemblies. The best assembler and polishing scheme were then validated to assemble the complete genomes of non-reference routine human and non-human samples.

### 2.1 Human whole-genome sequence datasets

#### 2.1.1 Human data for benchmarking of the assembly pipelines and polishing schemes

For the evaluation of all selected assembly and polishing tools, data from the HG002 sample (or NA24385, Son of Ashkenazi Jewish ancestry) was selected as reference since it is commonly used for calibration, development of genome assembly methods, and laboratory performance measurements as part of the GIAB Consortium. The raw Illumina and ONT sequence data from this sample is publicly available [30] (Table 1). Briefly, the Illumina dataset was obtained by sequencing on a NovaSeq 6000 System (Illumina, Inc.) and then downsampled to 35X, while the ONT dataset was obtained with PromethION (Oxford Nanopore Technologies) with R9.4 flow cells and base calling was performed using Guppy (v3.6.0), obtaining a genome coverage of 47X [27]. Note that the mitogenome scaffold was previously reconstructed using an in-house pipeline described elsewhere [31] and not assessed here.

**Table 1:** Summary information from human samples.

**Sample**	**Source of the genome**	**Sequencing technology**	**FASTQ generation**	Read length (bp)*	**Read depth (X)**	**Refs.**
HG002 (reference sample)	PrecisionFDA Truth Challenge V2 [24]	Illumina NovaSeq 6000	bcl2fastq (v2.20)	151	35	Olson *et al.* 2022 [27]
		ONT PromethION (R9.4 flow cells)	Guppy (v3.6.0)	50,380	47	
CAN0003 (validation sample)	CIRdb [26]	Illumina HiSeq 4000	bcl2fastq (v2.20)	151	29	García-Olivares *et al.* 2021 [31]
		ONT PromethION (R9.4 flow cells)	Guppy (v5.0.7)	17,227	37	

* N50 values were used to summarize read lengths for ONT.

#### 2.1.2 Human data for validation of the best performing pipeline

To validate the performance of the best pipeline resulting from the benchmarking, we used human whole-genome data from a routine sample from our laboratory (CAN0003) (Table 1). This sample was included in the reference genetic catalog of the Canary Islands population (CIRdb) that has been described elsewhere [32]. The details for column-based DNA isolation, library preparation, and sequencing were previously described [31]. This sample showed a DNA Integrity Number of 7.3, exhibiting a prominent and well-defined peak corresponding to high molecular weight DNA, indicative of limited fragmentation and overall high-quality integrity (Supplementary Table 1). Briefly, the short-read dataset was obtained using the Nextera DNA Library Preparation Kit and the sequence was obtained on a HiSeq 4000 Sequencing System (Illumina, Inc.) at the Instituto Tecnológico y de Energías Renovables (ITER, Santa Cruz de Tenerife, Spain). Raw BCL files were demultiplexed and converted to FASTQ files by means of bcl2fastq (v2.20). The ONT long-read dataset was obtained at Keygene (Wageningen, The Netherlands) using the ligation library preparation kit (SQK_LSK109) and sequenced on a PromethION platform (Oxford Nanopore Technologies) with a R9.4.1 flow cell (FLO_PR002) and MinKNOW (v1.14.2) software. After the run, base calling was performed with the Guppy (v5.0.7) neural network based tool. The mitogenome was obtained with the same methods as for the HG002 sample and was not assessed here.

### 2.2 Non-human sample datasets for pipeline validation

To evaluate the performance of the pipeline, genomes from different bacterial strains with differing GC content, *Campylobacter jejuni* (strain AS-83-79; GC: 30.5 %), *Streptococcus pneumoniae* (strain Jorgensen 262; GC: 39.5 %), and *Bordetella parapertussis* (strain 552; GC: 68 %), as well as from the *Herpes simplex* virus (strain MacIntyre; GC: 68.2 %), were sequenced and *de novo* assembled. The bacterial DNA was obtained from pure cultures, whereas the virus was obtained from infection of Vero cells. DNA from both the bacteria and the virus was extracted using the commercial TANBead solution (Taiwan Advanced Nanotech Inc.), with the TANBead Nucleic Acid Extraction Kit for Gram Bacteria (61G) and the OptiPure Viral Auto Tube, respectively. Short-read libraries were prepared using the Nextera XT Library Preparation Kit (Illumina, Inc.), and long-read libraries were prepared using the Native Barcoding Kit 24 V14 (SQK-NBD114.24). Short-read sequencing was performed on a MiSeq system (Illumina, Inc.) using a 2 × 300 bp paired-end run including a 5 % PhiX control library as spike-in, while long-read libraries were sequenced on a GridION X5 platform (Oxford Nanopore Technologies) using an R10.4.1 flow cell (FLO-MIN114) and MinKNOW software (v24.06). Basecalling of long-reads was performed using Dorado Basecall Server (v7.4) in Super-Accuracy mode.

### 2.3 Bioinformatics workflows

#### 2.3.1 Overview

**Fig. 1:**
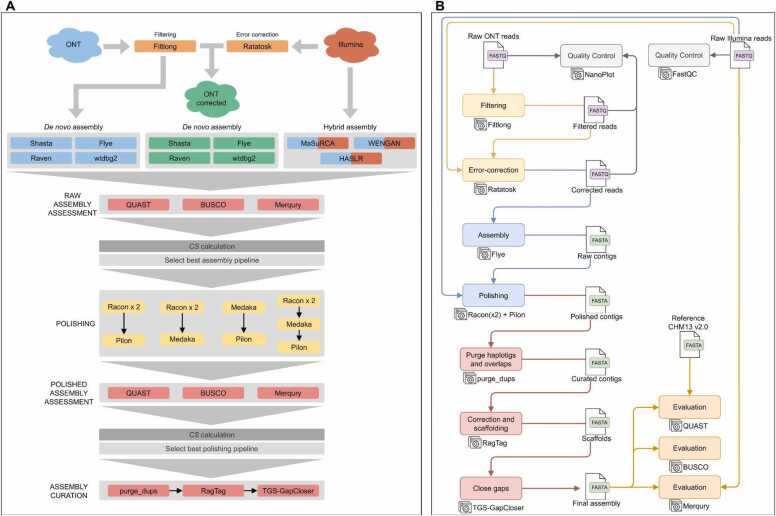
**A.** Long-read and hybrid *de novo* genome assembly pipelines for evaluation and benchmarking purposes using the reference sample data (HG002) as input. The datasets used in each pipeline were represented by colors: Blue, long-reads from ONT; Red, short-reads from Illumina; Green, long-reads from ONT corrected with short-reads. **B.** Detailed pipeline implemented in Nextflow using best assembly and polishing tools to validate previous results with the CAN0003 sample.

The evaluation of long-read and hybrid *de novo* assembly bioinformatics tools and the polishing pipelines were implemented using command-line interface Bash scripts (Fig. 1**A**). Based on the results, the complete analysis pipeline for the assembly, polishing, and contig curation using the best resulting tools (Fig. 1**B**) was developed on Nextflow (v23.04.1) [33] workflow management language using the templates and following the best practice guidelines provided by nf-core community [34]. This implementation enables efficient parallelization and built-in dependency management through Docker [35] containers and Conda [36] environments. Detailed usage of each bioinformatics tool and the complete pipeline is described in a dedicated repository: https://github.com/genomicsITER/hybridassembly.

#### 2.3.2 Filtering, error correction, and initial quality control steps

First, Filtlong (v0.2.1) (https://github.com/rrwick/Filtlong) was used to remove long-read sequences shorter than 1000 bp length from the ONT dataset. A base level error-correction step was performed afterwards using Ratatosk (v0.9.0) [37] to correct previous filtered long-reads using Illumina short-reads from the same sample.

Quality control assessments of ONT reads for the reference and validation sample datasets were performed using NanoPlot (v1.39.0) [38] before and after filtering and error correction processes. FastQC (v0.12.1) [39] was used to evaluate raw short-read sequence data.

#### 2.3.3 De novo genome assemblers benchmarked in this study

We evaluated the performance of four bioinformatics tools for *de novo* genome assembly of long and complex genomes using long-reads from ONT: Shasta (v0.9.0) [28], Flye (v2.9) [40], Raven (v1.8.1) [41], and wtdbg2 (v2.5) [42]. For these, two datasets of the reference sample (HG002) were used as input: i) the filtered long-reads, and ii) the filtered and the Ratatosk-corrected long-reads. With this, it was possible to evaluate the performance of the *de novo* assembly tools using error-correction processes prior to the assembly steps. For simplicity, we will refer to the first set of assemblies as Shasta, Flye, Raven, and wtdbg2, and to the second set as Corrected_Shasta, Corrected_Flye, Corrected_Raven, and Corrected_wtdbg2. All these assembly tools were run using default parameters.

We also evaluated the performance of the following three hybrid *de novo* assemblers: MaSuRCA (v4.0.8) [43], WENGAN (v0.2) [44], and HASLR (v0.8a1) [45]. For these, we used the reference sample dataset combining Illumina short-reads with the filtered ONT reads, and used default software parameters.

All assembly tools were executed using default parameters to ensure consistency, reproducibility, and fairness in future benchmarking studies.

#### 2.3.4 Assessment of raw genome assemblies

The quality of all resulting assemblies generated by the combination of assemblers and polishing tools was evaluated by means of QUAST (v5.0.2) [46], [47] using T2T-CHM13v2.0 as reference genome since it represents the first truly complete and gapless human genome assembly, covering all chromosomes, including previously unresolved centromeric and telomeric regions [48]. Its high accuracy and completeness make it an ideal benchmark for evaluating the continuity, correctness, and completeness of *de novo* assemblies. BUSCO (v5.3.2) [49], [50] using lineage primates_odb10 (2021-02-19), bacteria_odb10 (2024-01-08), and alphaherpesvirinae_odb10 (2024-01-08) databases were used to evaluate gene completeness of human, bacterial, and viral assemblies, respectively. In the human dataset used for the benchmarking, we also tested OMArk (v0.3.1) [51] to have a head-to-head comparison with BUSCO metrics. Merqury (v1.3) [52] was used as a reference-free assessment tool, to determine their quality using short-read sequencing data.

For each independent assembler, several metrics from QUAST, BUSCO, and Merqury were integrated in the Comprehensive Score (CS) calculation to comprehensively evaluate the quality of the assembled genomes provided by the assemblers and pipelines. This score was calculated similar to what has been described elsewhere [53], although including one additional metric provided by Merqury (further details in the Supplementary material). Briefly, CS integrated six metrics: the number of contigs, N50 in Mbp, number of mismatches and indels per 100 kbp, number of complete genes (completeness) annotated by BUSCO, and the consensus quality value (QV) estimated by Merqury.

#### 2.3.5 Comparing alternative polishing tools in the raw assembly with the best CS

The combination of input datasets, preprocessing steps, and assembly pipelines provided several possibilities. To simplify the comparisons of alternative polishing tools, we applied four schemes combining alternative state-of-the-art polishing tools, Racon (v1.5.0) [54], Medaka (v1.6.0) (https://community.nanoporetech.com), and Pilon (v1.24) [55], on the assembly with the best CS obtained in the previous stage. All polishing tools were executed using default parameters.

Four different polishing schemes were tested in order to assess the impact of running multiple rounds of polishing using only long reads or using both long and short reads (see Fig. 1**A**): a) two rounds of Racon combined with Pilon (referred as Racon_Pilon), b) two rounds of Racon combined with Medaka (referred as Racon_Medaka), c) one round of each Medaka combined with Pilon (referred as Medaka_Pilon), and d) all three combined with two rounds of Racon followed by one round each of Medaka and Pilon (referred as Racon_Medaka_Pilon).

Results of these polishing schemes were evaluated as for the raw assemblies, i.e., using QUAST, BUSCO, and Merqury to calculate the CS of the polished assemblies.

#### 2.3.6 Contig curation, scaffolding, and gap-filling

Polished contigs resulting from the polishing schema with best CS were curated using purge_dups (v1.2.6) [56] to remove haplotigs and contig overlaps based on read depth, reducing heterozygous duplication and increasing assembly continuity while maintaining completeness of the primary assembly. Potential misassemblies of curated contigs were further corrected and then ordered and oriented in the scaffolding step by means of RagTag (v2.1.0) [57] using the T2T-CHM13v2.0 as the reference genome. Gaps (Ns) present in the scaffolds were attempted to be filled using TGS-GapCloser (v1.2.1) [58].

#### 2.3.7 Validation of the pipeline with best results in a dataset from a routine human sample and in non-human sample datasets

Finally, in order to assess the performance of these tools in a real case scenario, we used data from a routine laboratory human sample with lower integrity than the reference material. To test the generalizability of the pipeline performance, we also used data from non-human datasets. The data from the validation samples were processed using the best assembly pipeline and polishing scheme, including curation, scaffolding, and closing-gaps steps (Fig. 1**B**). The final assemblies were assessed with QUAST, BUSCO, and Merqury.

### 2.4 Computational time and memory usage

Some of the key aspects to take into account in the context of this benchmarking are the computational time and resources associated with the *de novo* genome assembly process [59]. The performance of each selected assembly tool and polishing pipelines in this study were evaluated in terms of computational efficiency and time consumption by monitoring each process to obtain the total time execution and the peak of memory consumption.

### 2.5 Hardware resources

All the bioinformatics processes were conducted in two settings: an HPC cluster infrastructure, namely the TeideHPC (described here: https://teidehpc.iter.es), and a local workstation running CentOS 7 with 2 Intel® Xeon® Platinum 8358 CPUs at 2.60 GHz and with 2 TB of RAM.

## 3 Results

### 3.1 Benchmarking results

#### 3.1.1 Initial quality control of the reference HG002 sample dataset

The Illumina HG002 dataset consists of 415 Mreads with 151 bp length, providing a genome coverage of 39X. The raw ONT HG002 dataset consists of 19.3 Mreads with a N50 value of 50.3 kbp and a mean read quality of 8 in the Phred scale (Table 2).

**Table 2:** Raw sequence and preprocessed data characteristics of the HG002 genome.

**Sequence technology**	Step	Number of reads	Mean read length (bp)*	Mean read quality#	Total sequence (bp)	Theoretical depth of coverage (X)
Illumina	Raw	415,086,209	151	35.6	125,356,035,118	35
ONT	Raw	19,328,993	50,380	8.0	160,738,743,121	47
	Filtered$	5,645,728	54,270	12.6	144,664,869,506	45
	Corrected&	5,645,728	54,851	19.4	146,154,008,292	46

* N50 values were used to summarize read lengths for ONT.

# In Phred scale.

$ Removing sequences shorter than 1000 bp length.

& Removing sequences shorter than 1000 bp length and correcting the reads with short-reads.

Although filtering out shorter reads implies a drastic reduction in the total number of reads (Table 2), the resulting dataset maintains 90 % of the represented sequences, raising the N50 from 50.3 kbp to 54.3 kbp and increasing the mean read quality from 8.0 to 12.6. With the read error-correction, the N50 value improved slightly, as well as the total the represented sequences and the genome coverage. However, a substantial improvement was observed in terms of read quality.

Both filtered and corrected ONT datasets were used as input data for those assemblers that only use long-reads to investigate the impact of error-correction in the resulting assemblies. For the hybrid assemblers, the filtered ONT and raw Illumina datasets were used as input datasets.

#### 3.1.2 HG002 assembly results

Using previously preprocessed ONT and Illumina datasets of the reference HG002 sample, a total of 11 pipelines (resulting from the combinations of preprocessing datasets and assembly tools) were benchmarked based on the metrics obtained with QUAST, BUSCO, and Merqury (see Table 3 and Supplementary Table 2).

**Table 3:** Summary of HG002 assembly results and Comprehensive Scores (CS) obtained for each preprocessing strategy and *de novo* genome assembly pipeline. Contigs, N50 length (Mbp), mismatches (per 100 kbp), and indels (per 100 kbp) were extracted from QUAST using T2T-CHM13v2.0 as reference. The best value for each metric is shown in bold.

**Assembly type**	**Assembler**	**Contigs**	**N50 (Mbp)**	**Mismatches**	**Indels**	**QV**	**Completeness**	**CS**
ONT only assemblies	Shasta	2167	41.22	191.76	106.98	32.70	12,258	0.65
	Flye	845	38.29	141.24	107.35	33.73	12,249	0.71
	Raven	**313**	32.50	151.74	115.62	32.57	12,133	0.65
	wtdbg2	8712	10.34	228.31	274.81	25.82	10,849	0.15
	Corrected_Shasta	1153	8.75	115.90	41.65	40.69	12,974	0.76
	Corrected_Flye	819	**41.59**	127.48	26.16	**47.84**	**13,196**	**0.96**
	Corrected_Raven	583	28.21	**97.40**	32.18	40.54	13,176	0.88
	Corrected_wtdbg2	18,052	3.74	255.29	62.51	30.95	12,586	0.32
Hybrid assemblies	WENGAN	1314	29.31	111.85	26.87	45.32	13,164	0.90
	MaSuRCA	792	16.66	136.12	**25.41**	43.95	13,186	0.82
	HASLR	6030	1.03	112.74	70.57	36.26	12,620	0.60

QV, the quality value from Merqury. Completeness corresponds to the number of complete genes annotated by BUSCO.

In terms of contiguity, Flye, Raven, Corrected_Flye, Corrected_Raven, and MaSuRCA had the least number of contigs (< 1000 contigs), wtdbg2 and Corrected_wtdbg2 being the options with more fragmented assemblies (8712 and 18,052 contigs, respectively). Shasta, Flye, and Corrected_Flye showed the highest N50 values, near 40 Mbp. In contrast, Corrected_Shasta, Corrected_wtdbg2, and HASLR obtained assemblies with N50 values under 10 Mbp. The largest contig was returned by Shasta (138.11 Mbp), followed by Corrected_Flye (109.82 Mbp), WENGAN (109.74 Mbp), Raven (109.44 Mbp), and Flye (108.41 Mbp). Despite this, the retrieved total length of each was similar, ranging from 2.73 Gbp to 2.93 Gbp. The exception was Corrected_wtdbg2, which returned a total length of 3.28 Gbp, possibly due to the extremely high number of contigs returned.

**Fig. 2:**
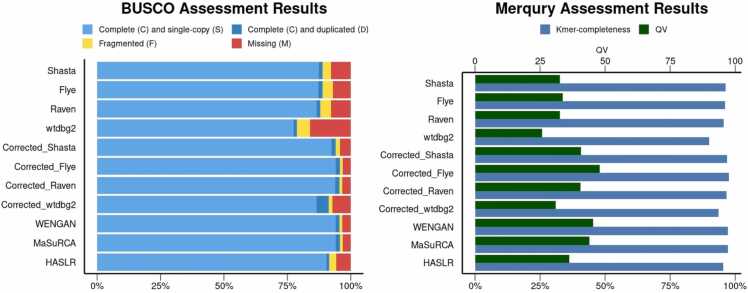
BUSCO and Merqury for HG002 assembly results for all 11 *de novo* genome assembly pipelines.

Regarding completeness, Shasta, Flye, and Corrected_Flye showed a NA50 value over 30 Mbp providing a genome fraction above 90 %. According to BUSCO, Corrected_Flye had the most complete gene number, closely followed by MaSuRCA, Corrected_Raven, and WENGAN, with more than 95 % of completeness reported by BUSCO. Based on Merqury metrics, Corrected_Flye, WENGAN, and MaSuRCA assemblers had the best k-mer completeness (> 97 %) and Quality Value (QV > 43) (Fig. 2). These results support that the combination with short-read sequencing data, either in the read error correction stage or at the assembly step, improves the *de novo* genome assembly.

Based on the correctness or accuracy of the assemblers, the best results were returned by Corrected_Raven, with 97.4 mismatches per 100 kbp, and Corrected_Flye, WENGAN, and MaSuRCA with nearly 25 indels per 100 kbp. The lower number of misassemblies were exhibited by HASLR (113), which also showed a small number of mismatches and indels, although with suboptimal results in terms of contiguity and completeness.

CS calculations for the 11 pipelines showed that Corrected_Flye and WENGAN were superior for *de novo* genome assembly combining ONT and Illumina data. However, Corrected_Flye provided the highest contiguity, resulting from a lower number of contigs (819) and a higher N50 value (41.59 Mbp). Corrected_Flye also had the best accuracy and completeness. Further details of the CS calculations can be found in the Supplementary Table 3.

Based on the previous results, the resulting assembly of Corrected_Flye was used in the following steps as the input to evaluate alternative polishing schemes. Overall, the polished assemblies show slight improvements in assembly quality. The schemes that used multiple rounds of Racon as a first polishing step showed greater improvements in contiguity, decreasing the number of contigs from 819 to 800, but maintaining the assembled size and N50. Both Racon and Medaka introduced more mismatches and indels that were subsequently corrected by the use of Pilon, resulting in a final improvement of the assembly correctness. However, in terms of completeness, the use of these polishing pipelines did not show differences, as the QV metric and the BUSCO completeness remained nearly identical in almost all situations, with the Racon_Medaka scheme being the one showing the worst results (see Table 4 and Supplementary Table 4). A comparison using OMArk as an alternative to BUSCO showed equivalent results (Supplementary Table 5).

**Table 4:** Summary of HG002 polishing results and CS values obtained for each scheme based on the best assembly (Corrected_Flye). Contigs, N50 length (Mbp), mismatches (per 100 kbp), and indels (per 100 kbp) were extracted from QUAST using T2T-CHM13v2.0 as reference. The best value of each metric is shown in bold.

**Polishing scheme**	**Contigs**	**N50 (Mbp)**	**Mismatches**	**Indels**	**QV**	**Completeness**	**CS**
Racon_Pilon	**800**	41.59	131.39	24.53	**46.58**	13,198	**0.65**
Racon_Medaka	**800**	**41.62**	133.99	28.02	42.68	13,196	0.33
Medaka_Pilon	819	41.59	**125.82**	**24.21**	45.77	**13,199**	0.63
Racon_Medaka_Pilon	**800**	41.60	127.93	24.57	44.21	13,196	0.56

QV, the quality value from Merqury. Completeness corresponds to the number of complete genes annotated by BUSCO.

Based on the CS values, the results revealed that Racon_Pilon was the best polishing scheme, closely followed by Medaka_Pilon. Further details underlying CS calculations can be found in Supplementary Table 6.

Finally, contig curation, scaffolding, and gap-filling steps were conducted using the polished draft assembly generated by the Racon_Pilon scheme. Resulting metrics from QUAST, BUSCO, and Merqury of the final curated *de novo* genome assembly are shown in Table 5. The complete assembly statistics of each curation step are shown in Supplementary Table 7.

**Table 5:** Summary statistics *de novo* genome assemblies of HG002 and CAN0003 before and after contig curation, scaffolding, and gap-filling steps. The T2T-CHM13v2.0 genome was used as the reference in QUAST evaluations.

**Tool**	**Metric**	**HG002 assembly**		**CAN0003 assembly**	
		**Raw**	**Curated**	**Raw**	**Curated**
QUAST	Total length (Gbp)	2.91	2.87	2.89	2.84
	# contigs	800	230	1,738	419
	N50 (Mbp)	41.59	144.97	26.38	143.46
	L50	21	8	30	8
	Largest contig (Mbp)	109.91	237.34	109.64	232.95
	Genome fraction (%)	92.30	91.58	91.53	90.39
	NA50 (Mbp)	31.63	44.66	22.30	32.77
	# misassemblies	3210	1571	2797	1362
	# Ns/100 kbp	0.00	38.03	0.00	182.54
	# mismatches/100 kbp	131.39	118.94	163.02	151.72
	# indels/100 kbp	24.53	23.94	31.30	30.55
Merqury	k-mer completeness (%)	97.49	97.32	97.42	97.06
	Quality value	46.48	47.65	42.46	42.92
BUSCO	Complete BUSCOs (C)	13,198	13,221	13,192	13,179

Before contig curation, the raw draft assembly consisted of 800 contigs with a total length at this stage of 2,91 Gbp, lacking any scaffolding or chromosome-level assignments, and the mitochondrial genome was not separated from the nuclear contigs. After curation, scaffolding, and gap-filling, the resulting assembly had 25 scaffolds, representing the 22 autosomes, X and Y sexual chromosomes, the mitogenome, and 206 unplaced contigs. Not considering the unplaced contigs, the total length of the assembly was 2,845,020,552 bp, including a total of 183 gaps summing up a total gap length of 1,092,630 bp. A detailed comparison of chromosome lengths and gaps between the curated assembled HG002 genome and the T2T-CHM13v2.0 reference can be found in the Supplementary Table 8.

#### 3.1.3 Computational time and memory usage

**Fig. 3:**
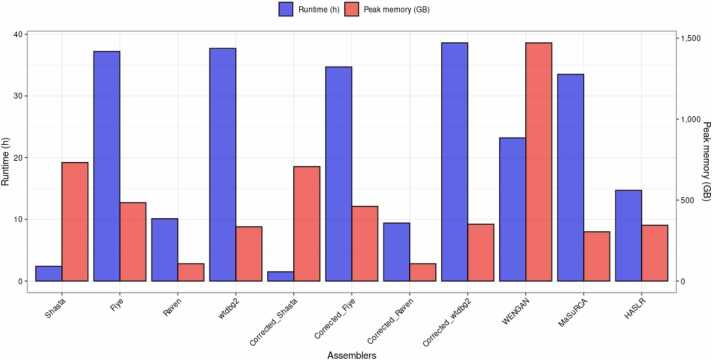
Computational resources (runtime and memory usage peak) required for the *de novo* genome assembly of HG002.

The computational resources required by each assembler in the local workstation setting were diverse, with computational runtimes ranging from 1.53 to 38.6 h, and a memory usage peak ranging from 107 to 1471 GB of RAM (Fig. 3). In terms of computational runtime, Shasta and Corrected_Shasta proved to be the fastest (2.4 and 1.5 h, respectively), although at the cost of a high (> 700 GB of RAM) memory usage peak. On the opposite, WENGAN was one of the tools with the highest computational costs both in terms of runtime (23.2 h) and memory usage peak (1471 GB of RAM). Raven and Corrected_Raven offered a situation of compromise, having a low resource intensity necessitating 107 GB of RAM, albeit still maintaining a reasonably low execution runtimes (10.1 and 9.4 h, respectively).

Regarding the polishing schemes, Racon_Pilon and Medaka_Pilon showed similar computational runtimes (42.1 and 41.3 h, respectively). In terms of memory usage, the Racon_Medaka_Pilon strategy, despite its longer computational time (60.4 h) maintains the same memory usage as Racon_Pilon (447 GB) due to the high memory cost of Racon. On the other hand, the Medaka_Pilon scheme has lower memory requirements, showing a peak memory RAM of 365 GB.

### 3.2 Assembly results of the human dataset for validation

#### 3.2.1 Initial quality control of the validation CAN0003 sample dataset

Multiple sequencing statistics including total sequenced reads and bases, average read length and quality, and the read length N50, were calculated for each dataset of this sample (Table 6). The Illumina dataset consisted of 341 Mreads with 151 bp length, providing a theoretical genome coverage of 29X. Raw ONT dataset consisted of 10.9 Mreads with a N50 value of 17.2 kbp and a mean read quality of 10.7. After filtration and correction steps, this dataset resulted in 7.5 Mreads with a N50 value of 18.3 kbp and a mean read quality of 18.4. As expected due to the laboratory handling and storage conditions of routine samples, a notable decrease in the number of reads and their overall shorter lengths were evident in comparison to those of HG002.

**Table 6:** Raw sequence and preprocessed data characteristics of the CAN0003 validation sample.

Sequence technology	Step	Number of reads	Mean read length (bp)*	Mean read quality#	Total sequence (bp)	Theoretical depth of coverage (X)
Illumina	Raw	340,531,948	151	36.6	93,116,844,148	29
ONT	Raw	10,928,576	17,227	10.7	118,191,926,169	37
	Filtered$	7,497,539	18,029	12.4	106,372,735,043	33
	Corrected&	7,497,539	18,287	18.4	107,891,043,566	34

* N50 values were used to summarize read lengths for ONT.

# In Phred scale.

$ Removing sequences shorter than 1000 bp length.

& Removing sequences shorter than 1000 bp length and correcting the reads with short-reads3.2.2 CAN0003 assembly results.

**Fig. 4:**
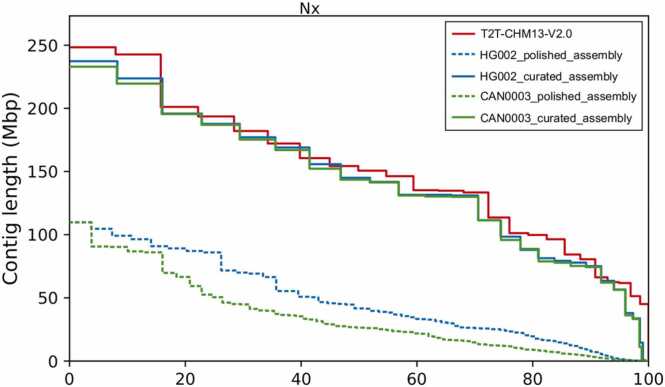
Nx plot of HG002 and CAN0003 polished and curated contigs resulting from *de novo* genome assembly using the Corrected_Flye pipeline and Racon_Pilon polishing schema, as reported by QUAST using T2T-CHM13v2.0 as the reference genome.

Based on the optimal performance on HG002, we used Corrected_Flye for *de novo* genome assembly and Racon_Pilon as the polishing scheme. The assembly results from QUAST, BUSCO, and Merqury of the final curated assembly are shown in Table 5. Further details of each curation step are shown in Supplementary Table 9. Compared to HG002, results for CAN0003 were similar in terms of contiguity (Fig. 4), showing more contigs (n = 419), although similar N50 values (143.46 Mbp) and a similar total length (2.84 Gbp). In terms of completeness, the results show 3.8 times more Ns/100 kbp (146.60 in contrast to 38.28 of HG002) and a similar number of complete BUSCOs (13,179). As for correctness, the CAN0003 genome assembly was worse than that of HG002, including 151.72 mismatches/100 kbp, and 30.55 indels/100 kbp.

The final curated assembly of CAN0003 had 25 scaffolds (1–22 autosomes, X and Y sexual chromosomes, and the mitogenome), plus 395 unplaced contigs. The total length of the assembly was 2,805,379,785 bp, not considering the unplaced contigs, and including 395 gaps summing up to a total gap length of 5,181,142 bp. A comparison of chromosome lengths and gaps between HG002 and CAN0003 genome assemblies, and the T2T-CHM13v2.0 reference is shown in Supplementary Table 8.

### 3.3 Results of the non-human sample datasets

To evaluate the generalizability of the benchmarked pipeline beyond human genomes, we applied the same workflow to additional non-human datasets, including three bacterial and one viral samples. These datasets feature considerably smaller genomes, allowing for a more straightforward assessment of assembly continuity and quality. In these cases, the pipeline successfully reconstructed highly continuous assemblies with high consensus quality and completeness, as confirmed by BUSCO scores (Table 7).

**Table 7:** Summary results of *de novo* genome assemblies of bacterial and viral samples.

**Tool**	**Metric**	***Bordetella parapertussis***	***Streptococcus pneumoniae***	***Campylobacter jejuni***	***Herpes simplex 1***
Sample information	Type	Bacteria	Bacteria	Bacteria	Virus
	Genome size (Mbp)	4.77	2.09	1.64	0.15
	Sequence used as reference (GenBank accession number)	CP025070.1	AP018938.1	AL111168.1	MN136523.1
QUAST	Total length (Mbp)	4.78	2.10	1.62	0.18
	# contigs	1	3	2	2
	N50 (Mbp)	4.78	2.09	1.58	0.14
	L50	1	1	1	1
	Largest contig (Mbp)	4.78	2.09	1.58	0.14
Merqury	k-mer completeness (%)	99.6	99.8	85.9	31.6
	Quality value	53.3	57.3	26.4	32.4
BUSCO*	Lineage dataset	bacteria_odb (2024-01-08)	bacteria_odb (2024-01-08)	bacteria_odb (2024-01-08)	alphaherpesvirinae_odb10 (2024-01-08)
	BUSCO groups searched	124	124	124	21
	Complete BUSCOs (C)	124	123	109	20

One exception was the viral dataset, where a complete assembly was not achieved due to the presence of non-isolated viral material, which likely introduced coverage biases and assembly fragmentation. These results highlight the robustness of the pipeline and its adaptability across diverse taxonomic groups and genome sizes.

## 4 Discussion

Given its unique capability to generate very long reads, nanopore sequencing data significantly influences the outcomes of *de novo* genome assembly compared to other sequencing technologies [48], [60], [61]. Despite the challenges posed by the higher per base error rates compared to other technologies [62], this benchmarking study highlights the potential of nanopore sequencing to span large genomic regions, contributing to enhance the contiguity in the results. For the first time, we compared up to 11 different pipelines for *de novo* genome assembly of human whole-genomes using nanopore reads from a reference GIAB sample, and up to four different polishing combination schemes. We found that the best performing combination, although not in terms of the required computational time and resources, was to filter out nanopore reads for a minimal size (< 1000 bp), correct them with short reads, and to run Flye assembler, followed by two rounds of Racon and one run of Pilon to polish the sequence with short reads. We then validated the results in sequencing datasets obtained from a routine laboratory sample, and therefore with suboptimal DNA integrity due to handling and storage conditions. The latter comparison provided a comparable number of scaffolds and a total length for the nuclear chromosomes, although having more unplaced contigs and sequence gaps than the reference materials. We also validated the performance of the best performing option in ONT R10 sequencing datasets to reconstruct bacterial and viral genomes, thus, empirically addressing the generalizability of the findings. Finally, we provide a Nextflow-based implementation of the complete pipeline providing this best performing option for *de novo* genome assembly to enable parallelization and dependency management by any user.

Diverse studies have provided comprehensive views of the advantages and limitations of the sequencing technologies and assembly methodologies across different datasets. For instance, Wick and Holt [63] found Flye as a highly reliable assembler on the basis of a benchmarking of eight long-read assemblers using prokaryote samples, although without assessing error-correction and polishing steps. Cosma *et al.* [64] reviewed several long-read *de novo* genome assemblers for ONT, PacBio CLR, and PacBio HiFi reads on diverse eukaryotic genomes using simulated and real data, also concluding that Flye was the best performing tool for ONT and PacBio CLR datasets. A recent study, focusing on the benefits of the multiple sequencing platforms instead of a benchmarking of the software tools, assembled two human samples from the GIAB Consortium comparing multiple sequencing datasets (PacBio CLR and HiFi, ONT, and Illumina short-reads), five *de novo* genome assemblers, and polishing strategies using either only long reads or using both short and long reads [65]. They concluded that PacBio HiFi was the best technology for genome assembly due to their high base quality, although Flye followed by polishing steps was recommended to assemble ONT reads. Our results are consistent with these previous studies, by presenting Flye as an assembly tool with high reliability for ONT data. However, it is worth mentioning that the pursuit of an optimal long-read assembler is shaped by factors such as the sequencing technology and genome complexity. It must be noted that the analyzes did not assess the performance of tools in genomic regions that are challenging to sequence, including medically relevant genes and highly repetitive sequences such as telomeres and centromeres in humans. Furthermore, we validated the results across diverse datasets, using not only one of the reference materials from GIAB but also from human and non-human routine laboratory samples, underscoring the reproducibility and optimal reliability of adopting a hybrid strategy based on different sequencing technologies [32], [48].

The polishing schemes that were evaluated in this study relied on Racon, Medaka, and Pilon, three state-of-the-art sequence polishing tools [18], [61]. These results demonstrate the importance of polishing draft assemblies for the construction of high-quality reference genomes by improving accuracy, assembly gaps, and potential assembly errors and misassemblies, as reported by recent studies. Chen *et al.* [66] studied the impact of polishing ONT-based bacterial assemblies with Illumina short-reads using two polishing tools. They concluded that NextPolish and, at least, two rounds of Pilon result in similar accuracy levels. As a particular case, Mc Cartney *et al.* [67] used Illumina and PacBio HiFi reads to apply accurate assembly corrections on the T2T-CHM13v0.9 human genome assembly, aiming to improve consensus accuracy, filling gaps, and fixing misassemblies [21]. Here, we opted to provide a comparison of various polishing tools and combinations, revealing that a Racon_Pilon scheme achieved a balanced improvement in contiguity and accuracy.

Interestingly, when base correction with Ratatosk and short-reads was performed, we observed an increase in the number of contigs generated by Raven and wtdbg2, contrary to the expected improvement in assembly contiguity. This counterintuitive result suggests that the compatibility between correction tools and assembly algorithms plays a significant role in overall performance. Ratatosk is designed to correct long reads using high-accuracy Illumina short-reads, which improves read-level accuracy but can also introduce biases in read structure or coverage, particularly in low-complexity or repetitive regions. Assemblers like Flye and Shasta, which are more robust to local inconsistencies, benefit from these corrected reads and produce more contiguous assemblies. In contrast, Raven or wtdbg2, which rely heavily on k-mer frequency, appear to be more sensitive to these changes in read characteristics, which may lead to difficulties in graph construction or repeat resolution, ultimately resulting in fragmented assemblies with more contigs. This observation underscores the importance of carefully evaluating the interactions between correction methods and assembly tools when designing hybrid assembly pipelines.

There are some limitations of this study. We have used data from two specific ONT basecallers (Guppy and Dorado) and two ONT flow cell versions (R9.4 and R10), but it is known that the development in this field by ONT is in continuous and rapid improvement [68], [69]. Therefore, future studies using more accurate ONT basecallers or flow cells could lead to significant improvements in genome assemblies. Additionally, in recent years, ONT protocols and reagents have been enhanced, enabling the production of ultra-long reads [70] and duplex reads [71]. These advancements are expected to improve accuracy and may impact the computational time necessities, which can make the use of error-correction or polishing steps using short-reads unnecessary [72]. The benchmarking of the assembly and polishing tools was primarily based on the HG002 reference sample, and while efforts were made to validate the best performing pipeline on a routine laboratory sample and in different non-human samples, differences in sample quality, DNA purity, and integrity could influence assembly results in other settings. Our approach is primarily focused on the benefits of combining ONT and Illumina technologies, providing a comprehensive evaluation of *de novo* genome assembly strategies. However, it is important to note that the inclusion of additional technologies, such as PacBio [29], optical mapping by Bionano Genomics [73], or other techniques such as Hi-C to leverage proximity regions to inform the assembly [23], could further enrich the diversity of genomic data and potentially enhance the overall quality of assembly outcomes [3], [74], [75]. Some of these other technologies offer unique advantages, such as higher base-level accuracy, improvements in the detection of structural variants, or refinements in phasing and scaffolding, and their integration into future studies could contribute to a more nuanced understanding of the individual differences in the genomic landscapes and the impact in disease. Our study, while insightful within the scope of ONT and Illumina, prompts future investigations to explore the synergies and optimizations by incorporating a broader spectrum of sequencing technologies.

Since we initiated this benchmarking analysis, several new tools have emerged that further advance the state of human genome assembly, particularly for long-read data. Notably, hifiasm [76] has demonstrated improved performance and accuracy [77]. This assembler includes a mode specifically optimized for ONT R10 flow cell reads and is increasingly recognized as a leading choice for ONT-based human genome assemblies. Additionally, Verkko [78], [79], developed by the T2T Consortium, represents a novel and highly effective assembler capable of producing chromosome-level assemblies by combining ultra-long ONT reads and PacBio HiFi data. While these tools were not available or mature at the time of starting our benchmarking, we acknowledge their potential impact and encourage future evaluations to include them. This rapid pace of development underscores the need for continuous benchmarking efforts to guide best practices in *de novo* human genome assembly.

The findings of this study underscore the benefit of integrating long-read sequencing, particularly from ONT platforms [70], with short-reads for hybrid *de novo* genome assembly of human whole genomes. Utilizing a combination of base-level error-correction tools, such as Ratatosk, and advanced assembly pipelines and polishers allowed us to obtain assemblies with high accuracy and completeness. Still, the observed differences in processing times and memory utilization among the tested pipelines emphasize the importance of selecting bioinformatics tools that adapt to the available computational resources and project timelines, especially in large-scale genomic studies. Our findings contribute valuable insights and guidance for researchers navigating the complexities of *de novo* genome assembly in diverse genomic contexts. Continuous algorithmic development, scalability optimization, and standardization efforts are essential for the evolving landscape of genomic studies, ensuring the adaptability and reliability of bioinformatics tools in deciphering complex genomes with unprecedented precision.

## CRediT authorship contribution statement

**Rafaela González-Montelongo:** Writing – review & editing, Resources. **José M. Lorenzo-Salazar:** Writing – review & editing, Visualization, Resources, Formal analysis. **Lourdes Ortiz:** Investigation, Resources, Writing – review & editing. **Carlos Flores:** Writing – review & editing, Writing – original draft, Supervision, Resources, Funding acquisition, Formal analysis, Conceptualization. **Pablo Mendoza:** Investigation, Methodology, Resources, Validation, Writing – review & editing. **Luis A. Rubio-Rodríguez:** Writing – review & editing, Software, Formal analysis, Data curation. **Adrián Muñoz-Barrera:** Writing – review & editing, Writing – original draft, Visualization, Software, Investigation, Formal analysis, Data curation, Conceptualization. **Almudena Corrales:** Writing – review & editing, Resources. **David Jáspez:** Writing – review & editing, Software, Formal analysis, Data curation. **Itahisa Marcelino-Rodriguez:** Writing – review & editing, Resources.

## Ethics statement

The study was approved by the Research Ethics Committee of the Hospital Universitario Nuestra Señora de Candelaria (CHUNSC_2020_95) and performed according to The Code of Ethics of the World Medical Association (Declaration of Helsinki).

## Declaration of Generative AI and AI-assisted technologies in the writing process

*During the preparation of this work the authors did not use any generative AI or AI-assisted* technologies *in the writing process. As such, the authors have manually reviewed and edited the content and take full responsibility for the content of the publication.*

## Funding

This research was funded by Ministerio de Ciencia e Innovación (RTC-2017-6471-1; AEI/FEDER, UE), co-financed by the European Regional Development Funds ‘A way of making Europe’ from the European Union; Cabildo Insular de Tenerife (CGIEU0000219140); by the agreements OA17/008 and OA23/043 with Instituto Tecnológico y de Energías Renovables (ITER) to strengthen scientific and technological education, training, research, development and innovation in Genomics, Epidemiological surveillance based on sequencing, Personalized Medicine and Biotechnology; and by Convenio Marco de Cooperación Consejería de Educación-Cabildo Insular de Tenerife 2021–2025 (CGIAC0000014697).

## Code and data availability

https://github.com/genomicsITER/hybridassembly.

## Conflict of interest

The authors declare no competing interests.
