# Supplementary material for: Benchmarking of bioinformatics tools for the hybrid de novo assembly of human and non-human whole-genome sequencing data
Source: Comput Struct Biotechnol J. 2025 Jul 13;27:3099–109. doi: 10.1016/j.csbj.2025.07.020 (PMC12284544; doi:10.1016/j.csbj.2025.07.020)
Supplement: Supplementary file 2 — Supplementary material [file mmc2.docx]

# Supplementary Material

## Comprehensive Score (CS) calculation

The CS score integrates metrics provided by: 1) QUAST: including contig numbers (*contigs*), N50 length in Mb (*N50*), the number of mismatches (*mismatches*), and indels per 100kb (*indels*); 2) BUSCO: including the number of complete genes evaluated (*completeness*); and 3) Merqury: in particular the consensus quality value (*QV*) taking advantage of the *k-mer* based assembly evaluation using Illumina reads.

These metrics were integrated into the following equation (1):

$SM=\frac{M -M_{min}}{M_{max}- M_{min}}$

where each metric (*M*) was scaled to [0, 1] by Min-Max normalization across different assembly or polishing pipelines and used to derive a Scaled Metric (*SM*). The *M_min_* or *M_max_* correspond to the minimum or maximum value of *M* among all results to be evaluated. Since high-quality assemblies are expected to have high *SM* in *N50*, *completeness*, and *QV* and low *SM* for *contigs*, *mismatches*, and *indels*, the following equation (2):

$rawCS=SM_{N50}+SM_{QV}+SM_{Completeness}-SM_{Contig}-SM_{Mismatches}-SM_{Indels}$

defines the Raw Comprehensive Score (*rawCS*) by summing across the six *SM* values, whose coefficients were set as 1 for the former three metrics and -1 for the latter three metrics to integrate the positive and negative contribution of each. Finally, to obtain the CS, the rawCS was rescaled to [0, 1] by Mix-Max normalization using the following equation (3):

$CS=\frac{rawCS -\left( rawCS_{min} \right)}{{rawCS}_{max}- {rawCS}_{min}}$

where *rawCS_min_* and *rawCS_max_* correspond to the minimum and maximum theoretical value of the *rawCS*, which can take the values of -3 and 3, respectively.

## References

[Zhang, Xue, Chen-Guang Liu, Shi-Hui Yang, Xia Wang, Feng-Wu Bai, and Zhuo Wang. 2022. “Benchmarking of Long-Read Sequencing, Assemblers and Polishers for Yeast Genome.” *Briefings in Bioinformatics*, March. https://doi.org/](http://paperpile.com/b/nXxMp9/yDc0)[10.1093/bib/bbac146](http://dx.doi.org/10.1093/bib/bbac146)[.](http://paperpile.com/b/nXxMp9/yDc0)
